# MPLA Case 2: A junior physicist attempts to improve radiotherapy workflow

**DOI:** 10.1002/acm2.13188

**Published:** 2021-03-19

**Authors:** Dongxu Wang, Gabbie Meis, Mary Gronberg, Cassandra Stambaugh, Leonard Kim

**Affiliations:** ^1^ Memorial Sloan Kettering Cancer Center New York NY USA; ^2^ The University of Iowa Iowa City IA USA; ^3^ University of Texas MD Anderson Cancer Center Houston TX USA; ^4^ Tufts Medical Center Boston MA USA; ^5^ MD Anderson Cancer Center at Cooper Camden NJ USA

**Keywords:** MPLA, leadership, professionalism, case study

## Abstract

This fictional case describes the challenging situation for a junior physicist, who joined her hometown's cancer center as a solo physicist after graduating from residency. She is concerned about providing optimal patient care as well as improving her work/life balance. She wonders how to move forward. The intended use of the case study, in either a facilitated learning session or self‐study, is to inspire the readers to discuss the situation, analyze the institutional and personal factors, apply relevant leadership skills, and propose action plans. This case study falls under the scope of, and is supported by, the Medical Physics Leadership Academy (MPLA). A sample facilitator's guide or self‐study guide is available upon request to the MPLA Cases Subcommittee.

Angela Rossi had been working as a solo physicist in Mississippi Rapids, Missouri for a few months now, but she still regularly ran into challenges in her position. The former physicist, Dr. John Samuelson, had just retired after training Angela for only a week. Angela wished the training could have been longer, but the cancer center administration did not want to pay for an extra full‐time physicist for her to work alongside. The one‐week overlap was already generous, they told her.

The Mississippi Rapids Cancer Center had two beam‐matched, state‐of‐the‐art linear accelerators (LINAC) for external beam radiation therapy and one high‐dose rate (HDR) brachytherapy afterloader for occasional gynecological brachytherapy procedures. Angela thought that the cancer center should create another physicist position, but they didn’t think so. Angela worried she was stretched too thin in her position. Maybe Dr. Samuelson was simply too good at handling all the physics duties in the past two decades. Although she initially thought of the demands of her position as an exciting challenge, the extended hours Angela spent learning about the equipment and ensuring proper patient treatments were beginning to take a toll on her mental health.

Despite her difficulties, Angela was grateful to have secured the physicist position in Mississippi Rapids, her hometown, when she, right out of her medical physics residency, was selected out of a pool of highly qualified applicants. Although physicist positions at cancer centers and hospitals across the nation were growing increasingly common, Angela had had her hopes set on the center down the road from her parents’ house and her childhood home. Her own father was treated at this center for prostate cancer a few years ago. Her parents helped take care of her two young children, and she felt blessed her children got to spend so much time with their grandparents.

Being a medical physicist as a young mother wasn’t easy though; Angela spent many early evenings performing quality assurance (QA) on the LINACs while keeping an eye on the clock, hoping to be able to make it home in time for dinner. And she had to rise early and report to work by 7:00 AM to ensure that morning warmup proceeded smoothly. Although her husband and parents were more than happy to take the kids to soccer practice and violin lessons, Angela often found herself wishing the six of them could all enjoy the family’s carbonara recipe around the dinner table together. Angela’s husband worked at home, providing IT support for a west coast company, so he was able to take on much of the morning routine with their kids. While he was as accommodating and understanding as could be, Angela still often felt a strong sense of guilt whenever she had to tell him at 4:00 or 5:00 PM that she would have to stay at work for an indeterminate amount of time, because, again, one of the two LINACs was down. He often worked until 7:00 PM, and if Angela worked late, he would be regularly interrupted by their persistent children, knocking on the office door to ask when Mommy was coming home or what the family was having for dinner.

Unfortunately, it was growing increasingly common to have a down LINAC at the cancer center. With her limited experience in troubleshooting simple LINAC faults, the times when the machine was “down” seemed to be growing exponentially. Angela regularly overheard hallway gossip about the machines and was worried the rest of the staff was judging her inexperience or even doubting her qualifications. She wished Dr. Samuelson had been able to train her for even just a week longer. Angela felt eager to prove herself and her abilities to the rest of the center.

Extended clinical hours due to a down LINAC could be caused by several factors though. For one thing, the combined patient load on the two LINACs was no more than 40 patients per day. Angela was sure even a single LINAC could handle all of them within a long day. With matched beams, this ought to be the thing to do, but Angela never saw the working LINAC taking patients from the down machine. Instead, the therapists assigned to the down LINAC got busy calling patients about delays, while the therapists on the working LINAC could get very idle at times yet show no sign of offering help to those on the down machine.

On one particular, not‐so‐busy day, when Angela had hopes of leaving work by 5 PM, one LINAC went down again around 3 PM. There were only six patients remaining on this machine. Angela thought the other LINAC could easily pick up the load. She went to ask the senior therapist on that LINAC, Linda, about the possibility.

In‐between patient treatments, Angela found Linda down the hall. The woman greeted Angela warmly, with a big smile.

“Hi dear,” began Linda, “I don’t have much time to talk before getting to my next patient, but let’s head to your office.”

Angela nodded, explaining the problem as they settled into chairs in the privacy of the physicist’s office: “With just six patients left, we should be able to transfer those scheduled on the down machine to the other LINAC. The machines are the same, so could we transfer the patients?”

Linda seemed to agree as Angela spoke, but shook her head. “I’m sorry, Angela, but that’s not how things are done around here. Maybe, just maybe, you can transfer patients, but how about therapists? Do you let one LINAC’s therapists go home?

“Our patients value individualized care,“ Linda continued, “in fact our ‘continuity of care’ is formally measured by cancer center administration. We therapists have incentives to maintain our list of patients. Plus, in such a small town, patients are comfortable with the therapists that they’ve always worked with before.”

Angela understood that point intimately. In Mississippi Rapids, Angela often ran into patients from the center at the grocery store and the pharmacy. “But,” she countered, “I’m sure patients would prefer to get home on time and keep their regularly scheduled appointments. I’m sure you all wouldn’t mind getting home on time either.” Angela added.

Linda laughed softly to herself, “Well, Angela, that’s just not how things are done. Therapists understand responsibility to our patients is more important than leaving on time.”

Angela tried not to take offense at the implied assumption that she was just trying to go home.

“In fact, and to be quite frank here,” Linda continued, “therapists are paid by the hour, and overtime rate is twice the regular rate. Quite a few therapists actually prefer staying longer.” The senior therapist stood up to leave, checking the clock as her next patient’s appointment time neared. “We really do want what’s best for our patients though, so if you have a different solution, I’d be more than willing to entertain the option. Unfortunately, with the way our performance is measured, we can’t transfer patients, in case we receive poor reports for our ‘continuity of care.’”

“Thank you for your time, Linda!” Angela called as the senior therapist bustled out of the room to get to her patient on time.

Surprised by the way the therapists’ care was assessed, Angela went to chat with Dr. Smith. The doctor had finished a busy day of consults, and Angela found him contouring in the Treatment Planning Room. Angela thought she could get some buy‐in from the doctor, because by state regulation, Dr. Smith couldn’t leave the office either until all radiation treatments were finished.

Not wanting to startle him or Ms. Hernandez, the dosimetrist sitting beside him, Angela knocked on the door frame. Pulling up a chair, Angela explained that one of the LINACs was down again, and if the current patients were transferred to the working machine, all treatments should be done by 5:00 PM.

Dr. Smith seemed distracted by contouring targets and only gave Angela a part of his attention until she mentioned leaving on time. He shrugged halfheartedly, turning towards the physicist. “Normally, I’m here doing *this*” he pointed back to the computer, “until late anyway.”

Angela nodded. She could only imagine what “late” meant.

Angela turned to Ms. Hernandez, who didn’t seem to take kindly to Angela’s interruption. Like Angela, Ms. Hernandez was hoping to leave on time, and she was waiting for Dr. Smith to finish contouring and approve a few plans. The dosimetrist knew that Dr. Smith’s quick asides could turn into long‐winded, hours‐long conversations about the practice. He had just started contouring when Angela walked in, and the dosimetrist was visibly just barely hiding her frustration towards the physicist.

Angela sensed this and was about to take her leave when Dr. Smith continued, “You will quickly come to realize that there are a lot of things in the radiation therapy workflow here that could be improved. I take issue with the therapists’ approach of divide and conquer. From what I’ve seen, even with three‐point patient setup that require quite a bit more manual labor, it is common to have only one therapist, usually the more junior one, moving patients while the other, usually the more senior one, sets up computer programs. I’ve heard the therapists say there’s simply too much to do at the same time. But I’ve seen a lot of large shifts, sometimes rotations, based on image guidance, indicating suboptimal patient setup. I have talked to the Chief Therapist about this before. ”

Angela had seen similar trends in therapists’ task division, but she had never heard complaints of unsatisfactory patient setup.

Dr. Smith continued again, “On the computer, I see a lot of notes written about the daily treatment, including who set up the patient, who loaded software, who aligned image and applied shifts, etc. These were said to increase accountability and reduce setup issues, but these notes take extra time to type. The therapists could have spent more time with the patients setting them up correctly instead of on the computer.”

Dr. Smith paused, glancing between the dosimetrist and Angela: “Honestly, the financial arrangement here states that I work for a physician practice group that the cancer center contracts from. So, even though I see that there could be improvements and may occasionally ask about issues I see, I really don’t think I have any authority over how the rest of the center’s employees work or operate, as long as they deliver the treatment correctly.” He turned back to contouring on the computer. “I’m sure there’s someone in the hospital side you can talk to about this.”

Just at this time, Angela’s pager rang; the LINAC engineer had arrived. Angela excused herself and went to greet the engineer.

As the engineer started working, Angela stayed in the vault to watch. She tried to focus on learning from the engineer, but she was distracted by her conversation with Dr. Smith. It became clear to Angela that she would need to go to hospital administration to discuss the idea of switching patients between machines. However, she feared her lack of experience put her at a great disadvantage in even raising the issue with management. What if shifting patients caused some error? Would she be fired because it had been her suggestion?

Angela remembered a rumor she heard about a previous dosimetrist. While it was unlikely to have been meant for the new hire’s ears, Angela overheard that a couple of years ago, the dosimetrist was fired because he had created a volumetric‐modulated arc treatment plan with a couch rotation, an unusual arrangement. The therapists were unaware of the couch rotation and had overridden the interlock when machine parameters did not match plan parameters. They ended up mistreating the patient, but the blame fell on the dosimetrist alone, costing him his job. Angela was shocked to hear this; she thought it was absolutely not fair to the dosimetrist. The therapists should have taken their share of responsibility for this incident. Angela speculated that the cancer center administrator being a former therapist had possibly biased management’s judgment. If this department had such a punitive environment, maybe she should just bear it at least until she got board‐certified next summer?

Angela also felt uncomfortable that Linda, the lead radiation therapist, hinted Angela wanted to change the status quo just because she wanted to go home! Angela couldn’t deny that she would prefer to leave on time, but she also thought the current arrangement was suboptimal patient care. Angela made patients her #1 priority during work hours but did feel there should be some work/life balance.

Now, well‐past 4:00 PM, Angela texted her husband that she would be late again that evening and asked her parents to make dinner for the family. Instead of the voice of her three‐year‐old excitedly telling her about the events of that day at preschool, Angela listened to the quiet whirring of the down LINAC. With multiple thoughts in her mind, Angela pondered what to do next.

## AUTHOR CONTRIBUTIONS

DW and GM drafted and revised the case text. MG, CS and LK made critical revision of the case text. MG, CS and LK wrote the sample facilitator’s guide. All authors have approved the final version.

## References

[1] Gronberg M , Wang D . Introduction to Medical Physics Leadership Academy (MPLA) Case Studies. J. Appl. Clin. Med. Phys. In Press. 10.1002/acm2.13187 PMC7984495 33739586

